# Valedictory of Dr. Bell

**Published:** 1847-02

**Authors:** 


*Valedictory of Dr. Bell.*—It is with unaffected regret, that we announce the retirement of Dr. John Bell from the editorial chair. The last number of the *Bulletin* contains his farewell. Dr. Bell, for the past quarter of a century, has been closely and honorably identified with the medical literature of this country. In extensive and varied learning, sound judgment, manly independence, agreeable and vigorous style—in a word, all the qualifications which unite to form the accomplished journalist, he has few equals. While we congratulate him upon the relief from editorial care which he contemplates with so much satisfaction, we feel that American periodical literature will, in his retirement, lose one of its most faithful and useful labourers.

The profession generally will unite with us in tendering our earnest wishes for his lengthened enjoyment of the *otium cum dignitate*, to which his valuable services have entitled him.

In relinquishing the wearisome task of journalising, however, he does not contemplate entire withdrawal from literary pursuits. He intimates his intention of preparing some works for the press, which, as the fruits of long experience and ripe scholarship, will doubtless add to his well-earned reputation.

